# Leukemia Inhibitory Factor Protects Neurons from Ischemic Damage via Upregulation of Superoxide Dismutase 3

**DOI:** 10.1007/s12035-015-9587-2

**Published:** 2016-01-09

**Authors:** Stephanie M. Davis, Lisa A. Collier, Christopher C. Leonardo, Hilary A. Seifert, Craig T. Ajmo, Keith R. Pennypacker

**Affiliations:** 1 Department of Molecular Pharmacology and Physiology, University of South Florida, 12901 Bruce B. Downs Blvd., Tampa, FL 33612 USA; 2 Department of Molecular and Cellular Physiology, Louisiana State University Health Science Center Shreveport, 1541 Kings Hwy, Shreveport, LA 77103 USA

**Keywords:** Stroke, Neuroprotection, Superoxide dismutase, Oxidative stress

## Abstract

Leukemia inhibitory factor (LIF) has been shown to protect oligodendrocytes from ischemia by upregulating endogenous antioxidants. The goal of this study was to determine whether LIF protects neurons during stroke by upregulating superoxide dismutase 3 (SOD3). Animals were administered phosphate-buffered saline (PBS) or 125 μg/kg LIF at 6, 24, and 48 h after middle cerebral artery occlusion or sham surgery. Neurons were isolated from rat pups on embryonic day 18 and used between 7 and 15 days in culture. Cells were treated with LIF and/or 10 μM Akt inhibitor IV with PBS and 0.1 % DMSO acting as vehicle controls. Neurons transfected with scrambled or SOD3 small interfering RNA (siRNA) were subjected to 24-h ischemia after PBS or LIF treatment. LIF significantly increased superoxide dismutase activity and SOD3 expression in ipsilateral brain tissue compared to PBS. Following 24-h ischemia, LIF reduced cell death and increased SOD3 messenger RNA (mRNA) in vitro compared to PBS. Adding Akt inhibitor IV with LIF counteracted the decrease in cell death. Partially silencing the expression of SOD3 using siRNA prior to LIF treatment counteracted the protective effect of LIF-alone PBS treatment. These results indicate that LIF protects neurons in vivo and in vitro via upregulation of SOD3.

## Introduction

Reactive oxygen species (ROS) play an instrumental role in both the acute and delayed phases of neuronal death during focal cerebral ischemia (FCI). During the early phase of ischemia, energy failure leads to membrane depolarization [1], calcium influx, and activation of pro-oxidant enzymes such as neuronal nitric oxide (NO) synthase [2] and nicotinamide adenine dinucleotide phosphate (NADPH) oxidase [3]. These enzymes generate ROS, such as peroxynitrite and superoxide, which damage cellular components and trigger neuronal death [4]. A second wave of neuronal death occurs during the period of delayed neuroinflammation, which may continue hours to days after the initial injury [5]. The activation of microglia exacerbates oxidative damage to neurons via inducible NO synthase activation [6]. Furthermore, matrix metalloproteinases derived from activated microglia compromise blood–brain barrier (BBB) function by disrupting tight junctions [7–9]. Increased BBB permeability allows for the invasion of peripheral lymphocytes, monocytes/macrophages, and neutrophils [10]. The accumulation of immune cells in the brain damages already degenerating neurons in the penumbra [11]. Since the therapeutic window for tissue plasminogen activator, the only FDA-approved drug for ischemic stroke, ranges from 3 to 4.5 h after stroke, it cannot target this delayed phase of neuronal death [12]. Consequently, the need for novel stroke therapeutics that will protect vulnerable neurons from oxidative stress remains high.

While small molecule scavengers of ROS have demonstrated limited efficacy in clinical trials [13], overexpressing endogenous antioxidant enzymes may be more effective than administering exogenous agents. These enzymes include those in the superoxide dismutase (SOD) family, which react with excess superoxide to form hydrogen peroxide and water. Three SOD isoforms have been identified in mammals [14, 15]. Moreover, a decreased activity of SOD enzymes yields deleterious effects on nervous system function. The G93A mutation in SOD1, a ubiquitously expressed cytoplasmic enzyme, is associated with familial amyotrophic lateral sclerosis (ALS) [16–18]. Dysfunction of SOD2, which regulates ROS production in the mitochondria [19], is implicated in the development of conditions such as Alzheimer’s disease [20–22] and Parkinson’s disease [22]. Superoxide dismutase 3 (SOD3), the extracellular isoform with the lowest expression in neural cells [23], opposes the negative effects of hypertension in the brain by increasing NO bioavailability and regulating cerebral blood flow [24].

Several murine studies have implicated the neuroprotective role of SOD enzymes following FCI. Delivery of the SOD1 gene via herpes simplex virus protects mice against cerebral ischemia/reperfusion injury [25]. SOD2 homozygous knockout mice had greater infarct volumes after transient FCI compared to their wild-type counterparts [26]. Conversely, mice overexpressing SOD3 showed resistance to neural cell damage following transient FCI [27]. Nonetheless, information regarding the role of SOD3 in protecting neurons remains limited.

Few studies focus on exogenous agents that upregulate the expression of these enzymes. Previous data from this lab demonstrates the therapeutic potential of upregulating endogenous antioxidant enzymes as a protective strategy against stroke. Specifically, the Akt-dependent upregulation of the antioxidant enzyme peroxiredoxin IV (Prdx4) contributes to human umbilical cord blood (HUCB) cell-mediated protection of oligodendrocytes [28, 29]. This lab has chosen to focus on released factors such as leukemia inhibitory factor (LIF), which provide neural cell protection while avoiding cellular treatment [28, 30].

LIF, an anti-inflammatory cytokine in the interleukin-6 family [31], binds to a heterodimeric receptor consisting of the LIF receptor (LIFR) and glycoprotein 130 (gp130) subunits. Upon binding, several downstream signaling pathways are activated including the MAPK [32], PI3K/Akt [33], and JAK/STAT pathway [34, 35]. These signaling cascades, especially the PI3K/Akt pathway, play an instrumental role in the pro-survival and neurotrophic effects of LIF [36].

LIF has shown promising results as a therapeutic in several animal models of neurodegenerative disease. The administration of LIF reduces demyelination in the experimental autoimmune encephalomyelitis model of multiple sclerosis [37] and a murine model of spinal cord injury [38]. In addition, LIF reduced degeneration of motor neurons in the SOD1 G93A murine model of familial ALS [39]. Recently, we showed that LIF decreases infarct volume, improves functional recovery, and upregulates Prdx4 in oligodendrocytes after ischemia [40]. Although LIF is released by several cell types during brain injury [41–43], its potential as a stroke therapeutic has not yet been determined.

The ability of LIF to activate protective pathways and reduce neural cell damage in several animal models of disease makes it a prime candidate for targeting delayed neuronal death after stroke. The goal of this study is to identify the molecular mechanisms by which LIF exerts neuroprotection after permanent FCI. This lab tested the hypothesis that LIF increases neuronal survival after stroke by upregulating SOD enzyme expression and activity.

## Materials and Methods

### Animal Care

All animal procedures were conducted in agreement with the NIH Guide for the Care and Use of Laboratory Animals. Experimental protocols were approved by the Institutional Animal Care and Use Committee at the University of South Florida. Power analysis was conducted prior to experiments to determine the minimum number of animals required to detect significant effects. Sprague–Dawley rats were purchased from Harlan Labs (Indianapolis, IN, USA), maintained on a 12-h light–dark cycle (07:00–19:00 hours) in a climate-controlled room, and allowed access to food and water ad libitum. Neurons for in vitro experiments were taken from embryonic day 18 (E18) rat pups obtained from timed pregnant dams. Young, male rats weighing 300 to 350 g were used for in vivo experiments.

### Permanent Middle Cerebral Artery Occlusion and Administration of LIF

Induction of FCI was achieved using the permanent middle cerebral artery occlusion (MCAO) model as previously described [44]. Briefly, an incision was made near the sternum and blunt dissection was used to expose the common carotid artery. After ligating and cutting the external carotid artery, a 40-mm monofilament was introduced and fed through the internal carotid artery. The monofilament was advanced to the base of the middle cerebral artery. The monofilament was secured and the wound was closed using polypropylene sutures. Laser Doppler (Moore Lab Instruments, Farmington, CT) was used to confirm the reduction in cerebral blood flow. Animals experiencing less than a 60 % reduction in CBF were excluded from analysis. For the sham MCAO procedure, the common carotid artery was exposed without subsequent occlusion of the middle cerebral artery. Animals were administered recombinant human LIF (ProSpec, Ness Ziona, Israel) (125 μg/kg) intravenously at 6, 24, and 48 h post-MCAO. Phosphate-buffered saline (PBS) pH 7.4 was used as a vehicle control for the LIF. Rats were euthanized 24, 48, and 72 h post-MCAO for immunohistochemistry or biochemical analysis. The number of subjects in each treatment group was as follows 24 h PBS: *n* =3; 24 h LIF: *n* = 5; 48 h PBS: *n* = 4; 48 h LIF: *n* = 4; 72 h PBS: *n* = 12; and 72 h LIF: *n* = 10.

### Histochemical Analysis

Tissue sections were prepared for histochemical analysis as previously described [45]. Following euthanization, animals were perfused transcardially with normal saline followed by 4 % paraformaldehyde in PBS (pH 7.4). Brains were removed and incubated in 4 % paraformaldehyde followed by 20 and 30 % sucrose solutions. Brains were cut into 30-μm sections including the bregma −1.7 mm through +3.3 mm. 3,3-Diaminobenzidine (DAB) immunohistochemistry was performed as previously described [46]. This procedure was also performed as previously described [28] on 18-mm glass coverslips containing primary cortical rat neurons. The following antibodies were used for immunological detection: mouse α-SOD3 (1:250; Novus Biologicals, Littleton, CO), rabbit α-phospho-Akt (Ser473) (1:50; Cell Signaling, Danvers, MA) [29], and rabbit α-active caspase-3 (1:1000; Sigma-Aldrich, St. Louis, MO) [29]. Biotinylated secondary antibodies (1:300; Vector Laboratories, Burlingame, CA) were used in conjunction with their complimentary primary counterparts.

Fluorescent immunohistochemistry was performed on brain tissue sections as previously described [28] using rabbit α-microtubule-associated protein 2 (MAP2) (1:200; EMD Millipore, Billerica, MA) and mouse α-SOD3 (1:250; Novus Biologicals) [47] antibodies. Tissue sections were also double-labeled with mouse α-SOD3 (1:250; Novus Biologicals) and rabbit anti-phospho-Akt (Ser473) (1:50; Cell Signaling) or rabbit-α-LIFR (1:200; Santa Cruz Biotechnology, Dallas, TX) [37] and rabbit α-MAP2 (1:200; EMD Millipore) [48] antibodies. AlexaFluor® 488 and 594 secondary antibodies (1:1000; Life Technologies, Carlsbad, CA) were used for fluorescent visualization and slides were mounted using VECTASHIELD® medium containing 4′,6-diamidino-2-phenylindole (DAPI) (Vector Labs). Images were captured using a Zeiss AxioCam color camera attached to a Zeiss AxioSkop2 microscope (Dublin, CA) interfaced with OpenLab imaging software (Boston, MA).

### Western Blot Analysis

Brain tissue was homogenized in whole cell lysis buffer and protein concentrations were measured using a Bradford Assay (Bio-Rad, Hercules, CA). Samples were prepared and run on SDS-PAGE gels (10 % for SOD3, 12 % for SOD2, and 15 % for SOD1). Nitrocellulose membranes were blocked in 5 % nonfat milk in TBS + 0.1 % Tween (TBST) for 1 h at 25 °C and probed with the following primary antibodies overnight at 4 °C: mouse α-SOD3 (1:500; Novus Biologicals) [47], rabbit α-SOD1 (1:500; Abcam, San Francisco, CA) [49], or rabbit α-SOD2 (1:500; Novus Biologicals) [50]. Membranes were then probed with AlexaFluor® 488 goat α-rabbit IgG (1:250) or AlexaFluor® 488 goat α-mouse IgG_1_ (1:250) for 1 h at 25 °C and visualized using a Typhoon 9410 Imager (GE Healthcare Life Sciences, Marlborough, MA). Membranes were also probed with mouse α-β-actin (1:5000; Novus Biologicals) [51] antibodies. All protein bands of interest were normalized to β-actin bands to act as a loading control.

### SOD Inhibition Assay

Prior to assay, brain tissue samples were diluted in an equal volume of dilution buffer to lower detergent concentrations. Percent inhibition of SOD activity was measured according to the manufacturer’s protocol (K-Assay, Seattle, WA). Absorbance was measured at 450 nm using a μQuant microplate spectrophotometer (BioTek, Winooski, VT) and KC Junior software (BioTek). The following equation was used to calculate % inhibition:

$$ \left[\left({A}_{\mathrm{Blank}\ 1}-{A}_{\mathrm{Blank}\ 3}\right)-\left({A}_{\mathrm{Sample}}-{A}_{\mathrm{Blank}\ 2}\right)\right]/\left({A}_{\mathrm{Blank}\ 1}-{A}_{\mathrm{Blank}\ 3}\right)\times 100\% $$

Average % inhibition rates for each set of sample dilutions were graphed using the nonlinear regression function. The slope of the linear portion of each standard curve was used to calculate the IC_50_ for each sample. The IC_50_ and protein concentrations of samples were used to calculate units of SOD activity per milligram protein lysate (U/mg).

### Cortical Neuronal Cultures

Rat primary cortical neurons were isolated as previously described [52]. Cells were counted using the Trypan Blue exclusion method and seeded at a density of 800,000 cells/well into 12-well plates coated with 0.1 mg/ml poly-l-lysine solution. Neurobasal complete media containing B-27 supplement and 1.25 mM glutamine (Life Technologies) was changed 24 h after seeding and subsequent media changes occurred on a weekly basis. Neurons were used between 7 and 15 days in vitro.

### In Vitro Ischemia

Oxygen glucose deprivation (OGD) was performed as previously described to induce in vitro ischemia [46]. Cells were given a fresh media change: 1 ml Dulbecco’s modified Eagle’s medium (DMEM) with 4.5 g/l d-glucose and sodium pyruvate per well for control cells and 1 ml DMEM without d-glucose or sodium pyruvate for cells subjected to OGD. Neurons were treated with either 50, 200, or 1000 ng/ml LIF. Sterile filtered PBS was used as a vehicle control for LIF. In another experiment, neurons were treated with PBS or 200 ng/ml LIF in the presence or absence of 10 μM Insolution™ Akt Inhibitor IV (EMD Millipore). This inhibitor blocks the ATP binding site of a kinase located upstream of Akt and downstream of PI3K. DMSO (0.1 %) was used as vehicle control for Akt Inhibitor IV. Following treatment with LIF and Akt Inhibitor IV, neurons were placed in an airtight chamber. This chamber was flushed with a gas mixture containing 94 % N_2_, 5 % CO_2_, and 1 % O_2_ (Airgas, Tampa, FL) for 15 min prior to sealing the chamber for 24 h. Following the 24-h incubation, cells were removed from the chamber and supernatants were collected to measure the release of lactate dehydrogenase (LDH). Cells were washed twice with PBS, pelleted via centrifugation, and snap-frozen. Pellets were stored at −80 °C for further biochemical analyses.

### LDH Assay

Neuronal death was quantified by measuring the release of LDH as previously described [46] using a colorimetric assay kit (Takara Biosciences, Madison, WI). Cell supernatant samples were diluted 1:4 in fresh medium prior to assay to avoid obtaining absorbance readings beyond the linear threshold. Absorbance was read at 490 nm using a μQuant microplate spectrophotometer (BioTek) and KC Junior software (BioTek). To quantify approximate levels of neuronal death, a standard curve was created as previously described [46] by lysing known quantities of neurons with 2 % Triton X-100 and measuring LDH release.

### Gene Silencing

Silencing the expression of SOD3 in cortical neurons was achieved via transfection with small interfering RNA (siRNA). This procedure was performed according to manufacturer’s protocol using a Nuclefector™ 2b Device and the Amaxa™ Basic Nucleofector™ Kit for primary mammalian neurons (Lonza, Allendale, NJ). Scrambled (Control-A) and siRNA sequences directed against the rat SOD3 gene were purchased from Santa Cruz Biotechnology. Freshly isolated cortical neurons were transfected with either scrambled or SOD3 siRNA using protocol G-013. Cells were removed from cuvettes and seeded into 12-well plates containing pre-equilibrated DMEM complete medium. Media was changed to Neurobasal Complete 24 h after seeding. Suppression of SOD3 expression was confirmed using quantitative reverse-transcriptase PCR (qRT-PCR).

### Two-Step qRT-PCR

Two-step qRT-PCR was performed as previously described with minor modifications [28]. AffinityScript® Master Mixes for reverse-transcriptase and qPCR reactions were obtained from Agilent Technologies (Santa Clara, CA). Synthesis of complementary DNA (cDNA) was performed per manufacturer’s instructions in a Mastercycler Gradient thermal cycler (Eppendorf, Hauppauge, NY). Reaction components were at 25 °C for 5 min to allow primers to anneal, 42 °C for 30 min to allow cDNA synthesis, and 95 °C for 5 min to quench the reaction. The concentration and quality of newly synthesized cDNA was determined using a NanoDrop ND-1000 spectrophotometer (Thermo-Fisher Scientific, Waltham, MA). Primer sequences for rat SOD3 and GAPDH genes were obtained from Qiagen (Valencia, CA). The qPCR reaction was performed according to manufacturer’s instructions using the Chromo4 DNA Engine thermal cycler and Opticon™ 3.1 Software (Bio-Rad) with the following steps: 15 min at 95 °C to heat the reaction mixture, 30 s at 95 °C to denature strands, 30 s at 55 °C to allow primers to anneal, and 30 s at 72 °C for strand elongation. This denaturing–annealing–elongation cycle was repeated 40 times. Following the qPCR reaction, melt temperature curve analysis was performed to detect the presence of primer–dimers in the reaction mixture. Cycle threshold values were determined by setting the threshold to a value where all samples were in the amplification phase.

### Data Analysis

All DAB immunohistochemical images were uniformly sharpened and converted to black and white using Jasc Paint Shop Pro 9 (Eden Prairie, MN). Optical density measurements for Western blot bands and histochemical staining was performed using ImageJ 1.49 (NIH, Bethesda, MD). All statistical tests were performed using GraphPad Prism 4.0 (La Jolla, CA) software and all data are expressed as the mean ± SEM. A Pearson-D’Agostino test was used to determine normality among data sets. Unless stated otherwise, experiments were analyzed via a one-way ANOVA followed by Fisher’s protected least significant difference post hoc test. Data with significantly different variances between treatment groups or a non-Gaussian distribution was analyzed via a Kruskal–Wallis *H* test. Mann–Whitney *U* tests were used to determine significance between pairs and a Bonferroni correction was applied to the *P* value based on the number of individual comparisons made. Statistical significance was determined using an alpha value set at *P* = 0.05. All *P* values reported are one-tailed.

## Results

### LIF Increases Total SOD Activity and SOD3 Expression 72 h Post-MCAO

Total SOD activity was measured in brain lysates from rats euthanized 24, 48, or 72 h after MCAO or sham surgery. Mean SOD activities were normalized to the mean activity in samples from 72 h sham-operated rats. Ipsilateral tissue samples from all MCAO rats demonstrated a time-dependent increase in SOD activity from 24 to 72 h, while activities in contralateral samples remained stable throughout this period. SOD activities were significantly altered among treatment groups at 72 h post-MCAO (*H* (3) = 13.29; *P* = 0.004). SOD activity was significantly higher in LIF-treated ipsilateral tissue compared to PBS-treated ipsilateral tissue (*U* = 1.00; *P* = 0.0237). In addition, there was a trend towards increased SOD activity in the PBS and LIF ipsilateral samples compared to their contralateral counterparts.

**Fig. 1:**
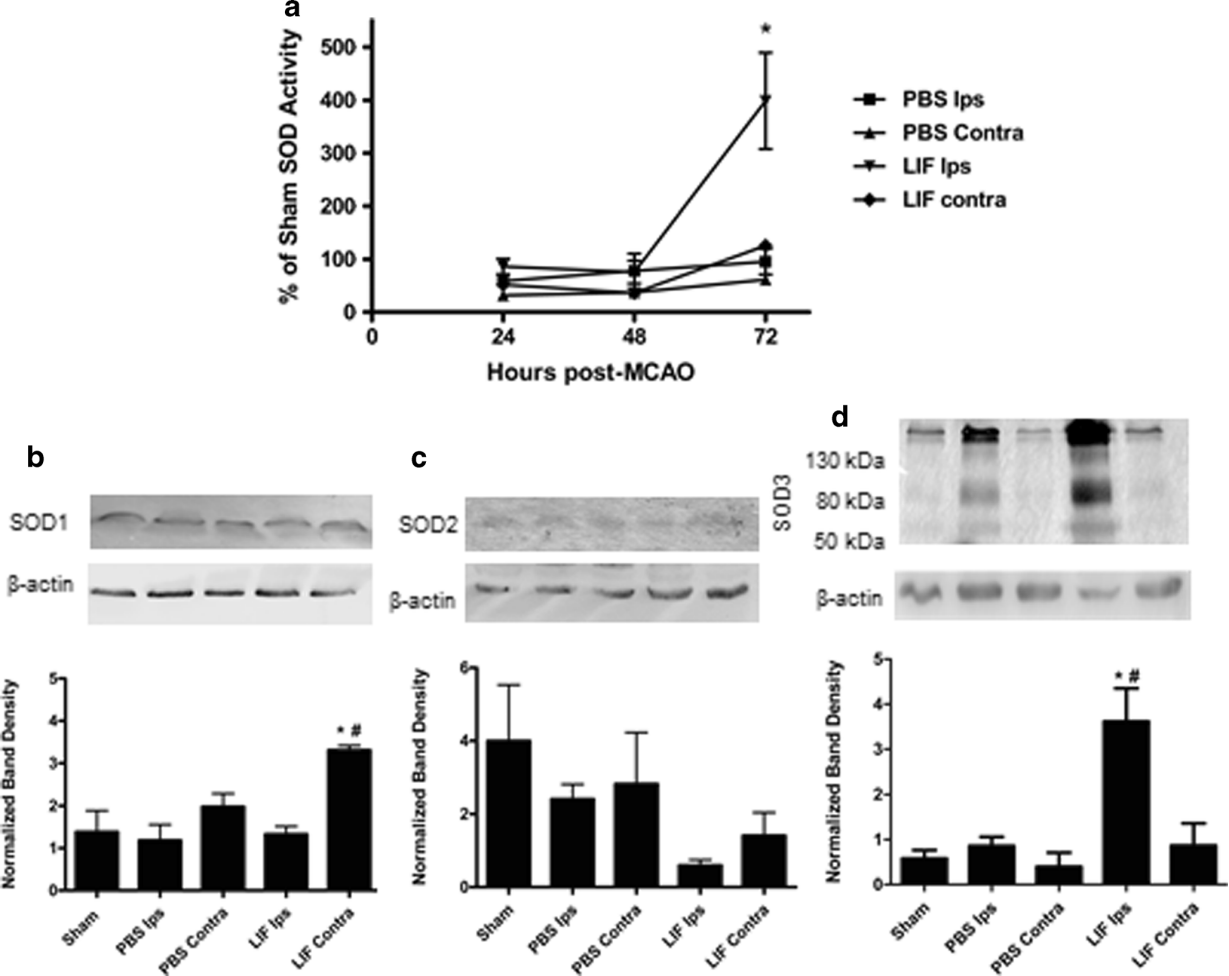
LIF increases SOD activity and SOD3 Expression. **a** Total SOD activity was measured in brain lysates from PBS- and LIF-treated rats euthanized 24, 48, or 72 h post-MCAO. LIF ipsilateral samples had significantly higher SOD activity compared to PBS-treated ipsilateral samples at 72 h post-MCAO (*P < 0.05). Mean activities were normalized to SOD activity in brains from sham rats. *n* ≥ 5 samples per group. **b** At 72 h after MCAO, SOD1 expression was significantly increased in contralateral tissue of LIF-treated rats, compared to ipsilateral tissue from PBS-treated rats (*P < 0.01) and ipsilateral tissue from the same treatment group (#*P* < 0.001). SOD1 bands were observed at approximately 18 kDa. **c** There was a trend towards decreased SOD2 expression in the ipsilateral samples from the LIF group; however, this decrease was not significant. SOD2 bands were observed at approximately 25 kDa. **d** Ipsilateral samples from LIF-treated rats showed a corresponding increase in SOD3 expression compared to PBS (*P < 0.05) and sham (#*P* < 0.01) ipsilateral samples. Three bands corresponding to SOD3 were observed in all samples at approximately 50, 80, and 130 kDa. *n* = 3 samples per group. *Ips* ipsilateral, *Contra* contralateral

Western blotting was used to determine protein expression of SOD1, SOD2, and SOD3 in brain tissue from rats euthanized 72 h post-MCAO. At 72 h post-MCAO, SOD1 was only significantly induced in contralateral tissue from LIF-treated rats (*P* = 0.0051, *F*_4,10_ = 7.321). Contralateral SOD1 expression from LIF-treated rats was significantly higher than that of ipsilateral tissue from PBS-injected rats (*P* = 0.0026) and ipsilateral tissue from the same treatment group (*P* = 0.0004; Fig. 1b). There were no significant differences in SOD2 expression between tissue samples at this time point (*H* (4) = 8.400; *P* = 0.0780), but there was a trend towards decreased SOD2 expression in ipsilateral tissue from LIF-treated rats compared to other samples (Fig. 1c). SOD3 expression was significantly higher in ipsilateral tissue from LIF-treated rats (*P* = 0.0021, *F*_4,10_ = 9.271) compared to ipsilateral tissue from PBS-treated rats (*P* = 0.0111) and sham-treated rats (*P* = 0.008; Fig. 1d). SOD3 expression remained relatively constant between the sham and contralateral samples.

**Fig. 2:**
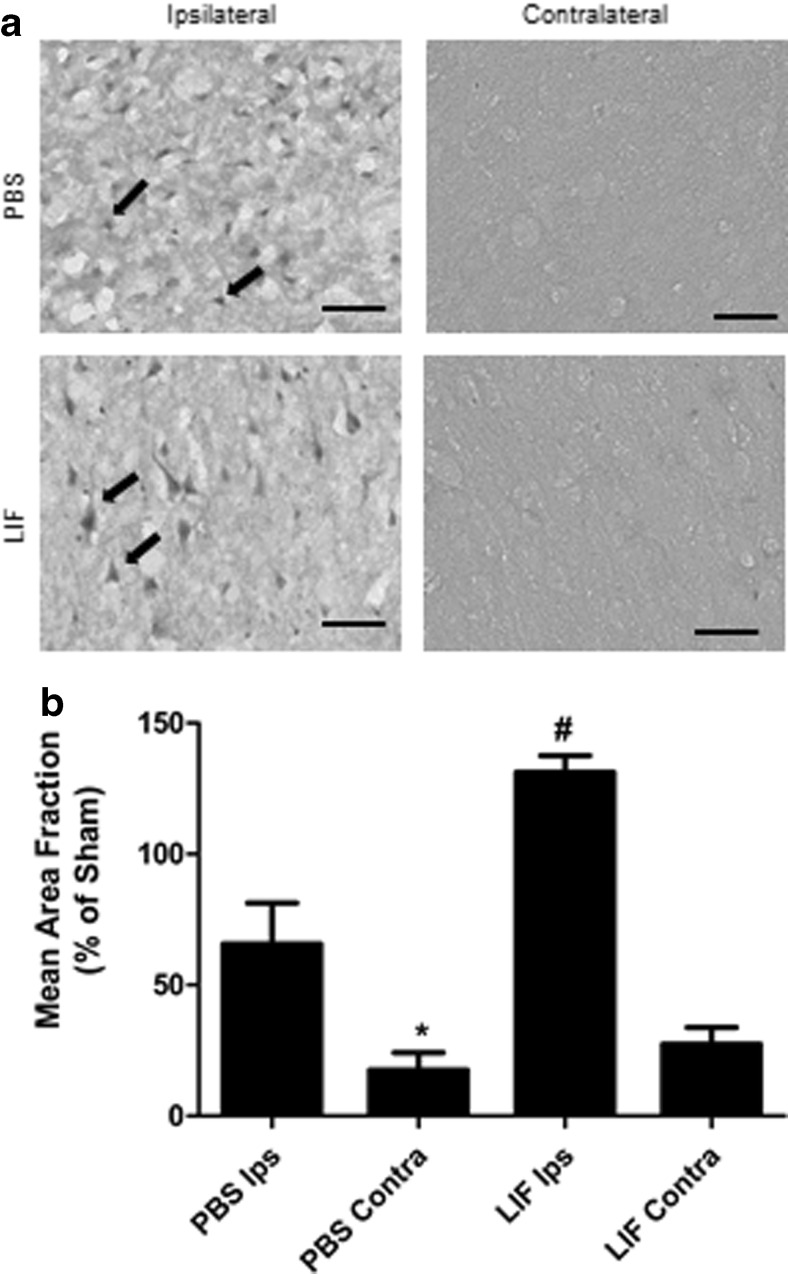
LIF upregulates SOD3 in the ipsilateral cortex. **a** Representative images of cortical tissue from PBS rats show higher levels of SOD3 staining compared to contralateral tissue from the same group. However, these cells appeared dysmorphic and unhealthy. LIF treatment increased SOD3 expression in the ipsilateral cerebral cortex while visibly reducing damage to SOD3-positive cells and surrounding tissue. **b** After normalizing to sham SOD3 levels, immunohistochemical quantification revealed significantly higher levels of staining in ipsilateral tissue compared to contralateral tissue in PBS-treated rats (*P < 0.05). LIF treatment further raised SOD3 in the ipsilateral tissue compared to PBS (#*P* < 0.01). *n* = 5 per treatment group. *Scale bars* = 50 μm. *Ips* ipsilateral, *Contra* contralateral

Immunohistochemistry was used to visualize SOD3 expression within the intact cortical tissue. Representative images show several SOD3-positive cells in the ipsilateral cortex of PBS-treated rats. However, these SOD3-positive cells appeared dysmorphic (black arrows) and many cells were surrounded by noticeable tissue lesions. By contrast, stained ipsilateral tissue from LIF-treated rats revealed SOD3-positive cells that retained a common triangular morphology compared to those found in PBS-treated rats. These cells were arranged in an orderly fashion and less tissue damage was observed in the LIF-treated rat brains. Contralateral cortical staining from both treatment groups did not reveal noticeable SOD3 staining (Fig. 2a). Quantitative analysis of cortical staining revealed induction of SOD3 in the ipsilateral cortex following LIF treatment (*P* < 0.0001, *F*_3,15_ = 24.38). Among PBS-treated rats, elevated levels of SOD3 were observed in the ipsilateral cortex compared to the contralateral cortex (*P* = 0.0111). However, LIF treatment yielded a significantly higher SOD3 expression in the ipsilateral cortex compared to PBS treatment (*P* = 0.0048). Levels of SOD3 staining were normalized to sham tissue to account for baseline SOD3 expression in the ipsilateral cortex (Fig. 2b).

### SOD3 Is Induced in Cortical Neurons Following LIF Treatment

**Fig. 3:**
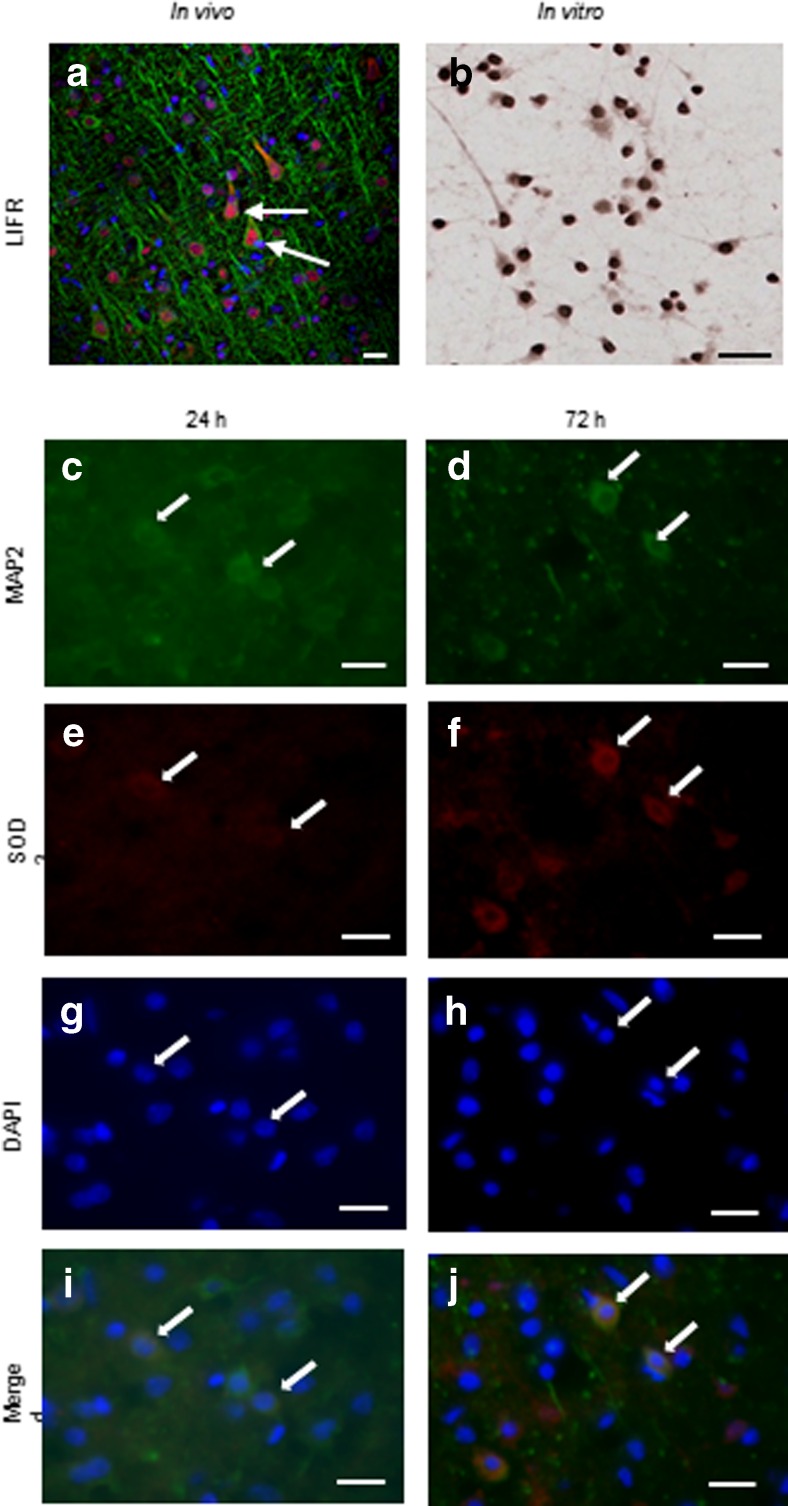
Cortical neurons express SOD3 after LIF treatment. **a** Co-labeling tissue with MAP2 (*green*) and LIFR (*red*) antibodies revealed localization of LIFR in the neuronal somata. LIFR was concentrated primarily in the cytoplasm and perinuclear region. **b** Cultures of cortical neurons also showed LIFR staining in the cell bodies. Following LIF treatment, neurons stained positive for MAP2 at **c** 24 h and **d** 72 h post-MCAO. SOD3 immunoreactivity was observed in the brains of LIF-treated rats at **e** 24 and **f** 72 h after MCAO. **g**, **h** DAPI was used to stain for cell nuclei. **i**, **j** Co-localization with the neuronal marker MAP2 indicates that SOD3 is expressed by neurons in vivo following LIF treatment. Arrows indicate representative cells. *Scale bars* = 20 μm

To determine whether LIF exerts positive neurotrophic effects on cortical neurons, brain tissue sections and cultured neurons were labeled with antibodies against LIFR and MAP2. Staining revealed localization of LIFR in neurons of the cerebral cortex (Fig. 3a). Cultured cortical neurons also expressed basal LIFR levels under physiological in vitro conditions (Fig. 3b). Tissue sections from LIF-treated animals that were euthanized 24 and 72 h post-MCAO were double-labeled with SOD3 and MAP2 antibodies. MAP2-positive neurons were observed at 24 and 72 h after MCAO (Fig. 3c, d). Likewise, SOD3-positive cells were also visualized at these time points (Fig. 3e, f). DAPI was used to indicate the presence of cell nuclei at both time points (Fig. 3g, h). Co-localization of SOD3 and MAP2 was observed at 24 and 72 h after MCAO, indicating the presence of SOD3-positive neurons at both time points (Fig. 3i, j).

**Fig. 4:**
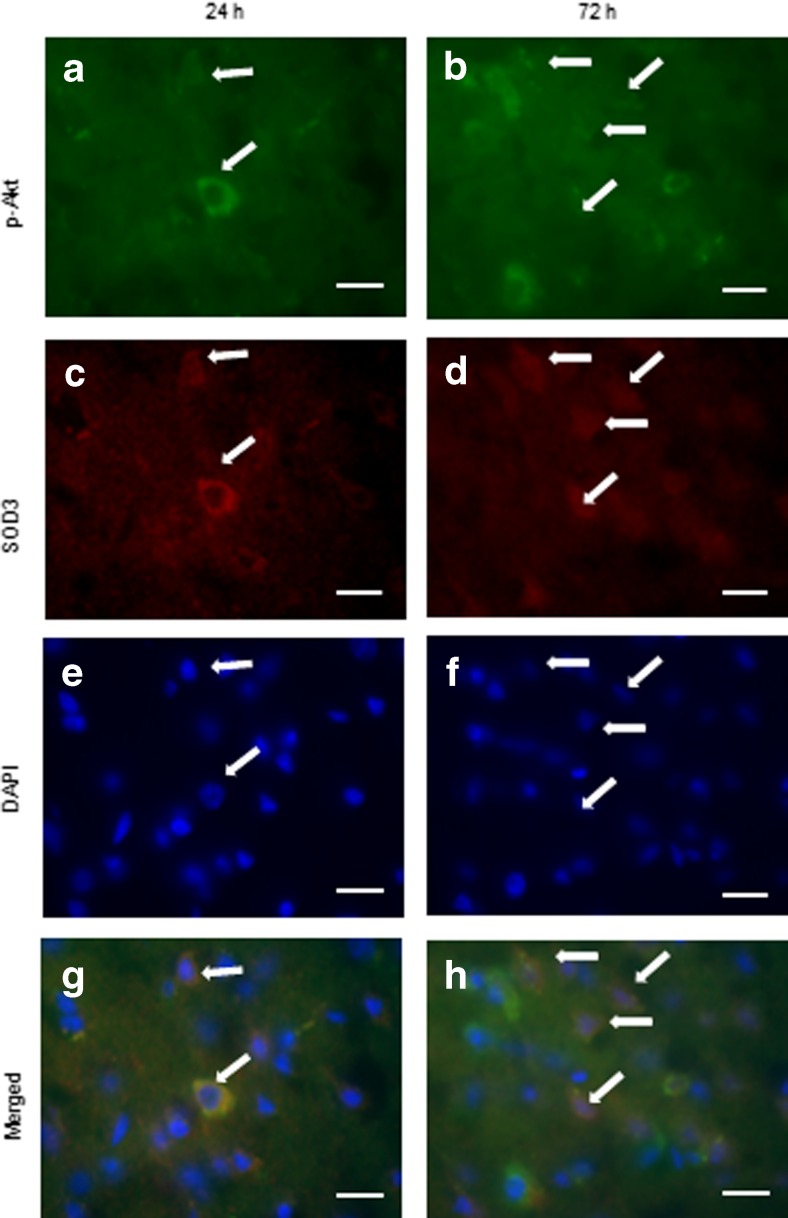
SOD3 co-localizes with phospho-Akt (Ser473). Levels of phospho-Akt (Ser473) increased from **a** 24 h to **b** 72 h after MCAO, which corresponded with increased SOD3 staining over this same time period (**c**, **d**). DAPI was used to stain for cell nuclei (**e**, **f**). The number of cells staining positive for phospho-Akt (Ser473) and SOD3 increased from **g** 24 h to **h** 72 h post-MCAO. *Arrows* indicate representative cells. *Scale bars* = 20 μm. *P-Akt* phospho-Akt (Ser473)

To determine whether this increase in neuronal SOD3 occurred in conjunction with Akt activation, tissue sections from LIF-treated rats euthanized at 24 and 72 h after MCAO were double-labeled with antibodies against SOD3 and Akt phosphorylated on residue Ser473 (phospho-Akt). Several cells stained positive for phospho-Akt at 24 and 72 h post-MCAO (Fig. 4a, b). Likewise, SOD3-positive cells were also visualized at both time points (Fig. 4c, d). Cell nuclei were labeled using DAPI at 24 and 72 h post-MCAO (Fig. 4e, f). Cells staining positive for phospho-Akt and SOD3 were observed at 24 and 72 h post-MCAO (Fig. 4g, h).

### LIF Protects Neurons from In Vitro Ischemic Damage

**Fig. 5:**
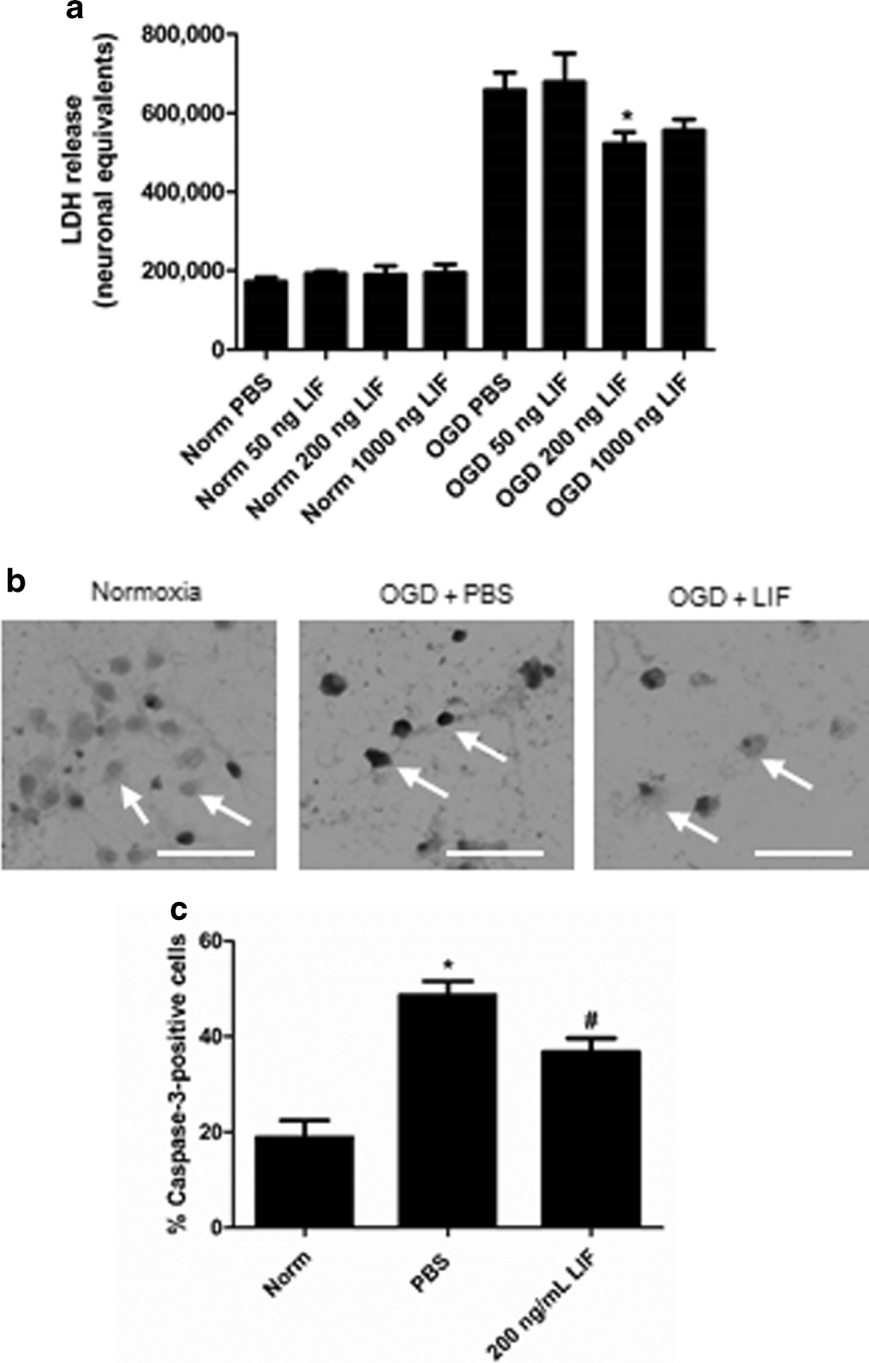
LIF treatment protects neurons in vitro against OGD. **a** Following 24 h OGD, 200 ng/ml LIF significantly reduced cell death compared to PBS treatment *(#P <* 0.05). *n* ≥ 3 per treatment group. **b** Images show neurons stained with α-active caspase-3 antibodies after 24 h normoxia, 24 h OGD + PBS, and 24 h OGD + 200 ng/ml LIF. *Arrows* identify representative cells. **c** Neurons treated with PBS prior to OGD had significantly higher levels of active caspase-3 (*P < 0.001) compared to the 24 h normoxia group. LIF treatment prior to 24 h OGD decreased caspase-3 activation compared to PBS (#*P* < 0.05). *Arrows* identify representative cells. *n* = 4 per treatment group. *Scale bar* = 50 μm. *Norm* 24 h normoxia

This laboratory previously demonstrated that 200 ng/ml LIF protects oligodendrocytes during OGD [40]. A similar concentration response experiment was performed to determine whether this concentration of LIF confers neuroprotection against in vitro ischemia. Subjecting neurons to 24 h OGD and treating with LIF yielded a significant change in LDH release (*H* (7) = 25.74; *P* = 0.0006) Treating cells with 200 ng/ml LIF significantly decreased LDH release compared to PBS-treated neurons that were subjected to the same 24-h OGD exposure (*U* = 7.000; *P* = 0.0256; Fig. 5a). To confirm the results of the LDH assay, neuronal cultures were subjected to the same conditions and treatments but were subsequently labeled with antibodies generated against activated caspase-3. Representative images show levels of active caspase-3 in neurons following 24 h normoxia, 24 h OGD + PBS, or 24 h OGD + 200 ng/ml LIF (Fig. 5b). Immunohistochemical analysis revealed significant changes in caspase-3 activation triggered by OGD exposure and LIF treatment (*P* = 0.0003, *F*_2,9_ = 23.57). Caspase-3 immunoreactivity was significantly higher after 24 h OGD + PBS compared to 24 h normoxia (*P* = 0.0003) while LIF treatment reduced caspase-3 activation during OGD compared to PBS treatment (*P* = 0.0128; Fig. 5c).

### Neuroprotective Effects of LIF Are Dependent upon Akt Activity

**Fig. 6:**
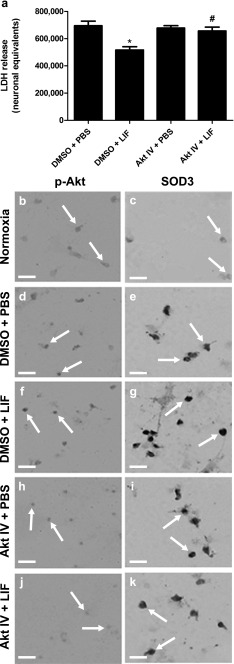
In vitro neuroprotection by LIF is dependent upon Akt activity. **a** LIF treatment significantly lowered neuronal death during OGD compared to PBS (*P < 0.001). **b** Akt IV reverses the neuroprotective effect of LIF treatment alone *(#P <* 0.01). *n* = 12 per treatment group. Representative images show *B* p-Akt and *C* SOD3 immunoreactivity in neurons under normoxia. DMSO + PBS treatment before 24 h OGD increased *D* p-Akt and *E* SOD3 levels compared to 24 h normoxia. *F* Phospho-Akt (Ser473) and *G* SOD3 levels were highest in cells treated with DMSO + LIF before OGD. Addition of Akt IV + PBS prior to OGD caused *H* a minimal decrease in phospho-Akt (Ser473) and *I* no change to SOD3 levels compared to the DMSO + PBS group. Co-incubation with Akt Inhibitor IV + LIF prior to OGD prevented the LIF-induced increase in *J* phospho-Akt (Ser473) and *K* SOD3 expression. *Arrows* identify representative cells. *P-Akt* phospho-Akt (Ser473), *Akt IV* Akt Inhibitor IV. *Scale bar* = 25 μm

To determine whether the protective effects of LIF are dependent upon Akt activity, neurons were treated with PBS + 0.1 % DMSO, 200 ng/ml LIF + 0.1 % DMSO, PBS + 10 μM Akt Inhibitor IV, or 200 ng/ml LIF + 10 μM Akt Inhibitor IV prior to OGD. LIF and Akt IV treatment both exerted significant effects on LDH release following 4 h OGD (*H* (3) = 17.55; *P* = 0.0005). Treatment with 200 ng LIF significantly reduced LDH release compared to PBS (*U* = 15.00; *P* = 0.0012). Co-incubating cells with 10 μM Akt Inhibitor IV and LIF yielded significantly higher levels of LDH compared to neurons treated with LIF alone (*U* = 19.00; *P* = 0.0024; Fig. 6a). To determine whether this decrease in neuronal death correlated with higher expression of SOD3 in vitro, representative coverslips subjected to 24 h normoxia or 24 h OGD with DMSO + PBS, DMSO + LIF, Akt Inhibitor IV + PBS, or Akt Inhibitor IV + LIF treatment were stained with antibodies directed against phospho-Akt or SOD3. Neurons incubated under normoxia revealed low basal levels of phospho-Akt and SOD3 (Fig. 6(B, C)). Cells treated with DMSO + PBS followed by 24 h OGD yielded higher levels of phospho-Akt and SOD3 compared to normoxic cells (Fig. 6(D, E)). However, neurons that were treated with DMSO + LIF prior to OGD showed high levels of phospho-Akt and SOD3 while returning to the healthy morphology seen in normoxic cells (Fig. 6(F, G)). Treatment with Akt Inhibitor IV + PBS prior to OGD decreased phospho-Akt compared to the neurons treated with DMSO + PBS (Fig. 6(H)), but SOD3 levels did not change (Fig. 6(I)). Co-incubation with LIF and Akt Inhibitor IV lowered phospho-Akt and SOD3 staining compared to neurons treated with DMSO + LIF (Fig. 6(J, K)).

### Neuroprotection Against OGD by LIF Is Dependent upon Increased SOD3 Expression

**Fig. 7:**
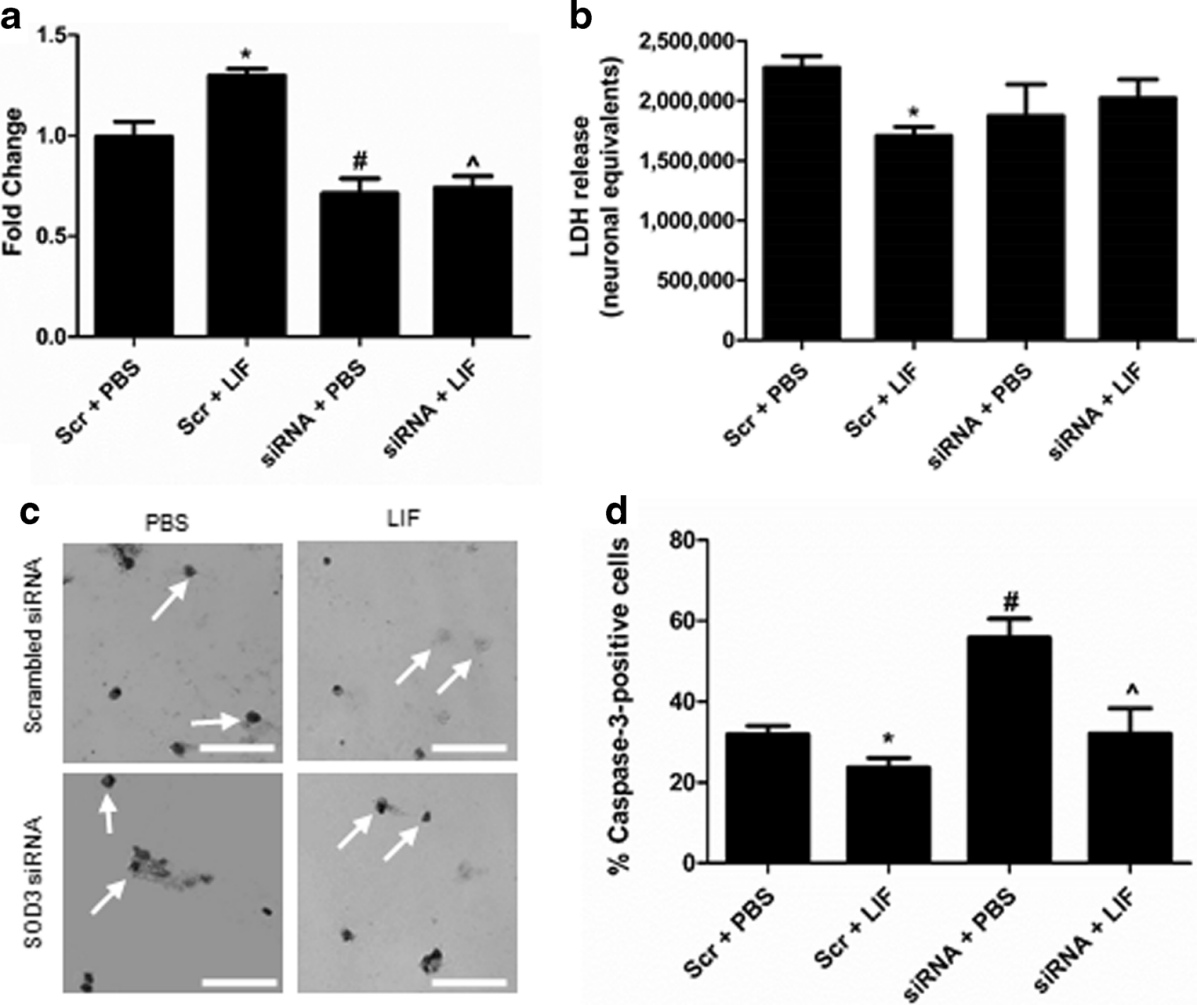
LIF increases cell survival by upregulating SOD3. **a** LIF treatment prior to 24 h OGD increased SOD3 mRNA among neurons transfected with scrambled siRNA (*P < 0.01). SOD3 siRNA treatment + PBS prior to OGD significantly decreased SOD3 mRNA compared to scrambled siRNA + PBS (#*P* < 0.05). SOD3 siRNA + LIF reduced SOD3 mRNA compared to scrambled siRNA + LIF (^*P* < 0.001) and scrambled siRNA + PBS. *n* ≥ 4 per treatment group. **b** LDH release was significantly lower in scrambled siRNA-transfected cells treated with LIF compared to their PBS-treated counterparts (*P < 0.05) while LIF treatment did not reduce LDH release in SOD3 siRNA-transfected cells compared to SOD3 siRNA + PBS cells. *n* = 3 per treatment group. **c** Representative cells show levels of active caspase-3 among treatment groups. **d** Active caspase-3 immunoreactivity was significantly reduced by LIF treatment in neurons transfected with scrambled siRNA compared to Scr + PBS cells (*P < 0.05). Transfection with SOD3 siRNA + PBS treatment significantly increased caspase-3 activation compared to Scr + PBS treatment (#*P* < 0.001). SOD3 siRNA + LIF cells had significantly lower levels of active caspase-3 compared to SOD3 siRNA + PBS cells (^*P* < 0.05), but not Scr + PBS cells. *n* ≥ 3 per treatment group. *Scr* scrambled siRNA. *Scale bar* = 50 μm

Transfection with siRNA and treatment with LIF prior to 24 h OGD significantly altered levels of SOD3 messenger RNA (mRNA) in primary cortical neurons (*P* < 0.0001, *F*_3,17_ = 17.51). LIF significantly increased SOD3 mRNA compared to PBS treatment in cells transfected with scrambled siRNA (*P* = 0.0023). Transfection with SOD3 siRNA significantly downregulated the expression of SOD3 compared to scrambled siRNA (*P* = 0.0165). Transfection with SOD3 siRNA prior to LIF treatment significantly decreased SOD3 expression compared to scrambled siRNA + PBS cells (*P* = 0.0212) and siRNA + LIF cells (*P* = 0.0005; Fig. 7a). LIF and siRNA treatment yielded an overall trend towards altered LDH release following 24 h OGD (*P* = 0.1701, *F*_3,8_ = 2.165). However, LIF treatment significantly reduced LDH release among neurons transfected with scrambled siRNA (*P* < 0.05; Bonferroni method; Fig. 7b). Representative images show levels of active caspase-3 among treatment groups (Fig. 7c). Treatment with LIF and SOD3 siRNA significantly altered caspase-3 activation following OGD (*P* = 0.0002, *F*_3,13_ = 14.50). LIF significantly reduced caspase-3 activation following OGD compared to PBS treatment among scrambled siRNA-transfected cells (*P* = 0.0104). SOD3 siRNA + PBS treatment prior to OGD significantly increased activation of caspase-3 compared to scrambled siRNA + PBS treatment (*P* = 0.0003). LIF significantly lowered caspase-3 activation compared to PBS among SOD3 siRNA-transfected cells (*P* = 0.0214). However, SOD3 knockdown prevented LIF from reducing caspase-3 activation compared to scrambled siRNA + PBS cells (Fig. 7d).

## Discussion

SOD3, unlike SOD1 and SOD2, is not highly expressed by neural cells under normal physiological conditions [23, 53]. Nonetheless, several distinct neuronal populations will upregulate SOD3 following FCI, including cortical and striatal neurons [54]. These data demonstrate that LIF treatment further increases SOD3 in neurons following ischemic injury. At 72 h post-MCAO, antibodies against SOD3 detected three Western blot bands corresponding to the monomer (40 kDa), dimer (80 kDa), and tetramer (140 kDa) of SOD3. Wild-type SOD3 is expressed as a dimer in rats; compared to the tetrameric human and murine isoforms, a single-point mutation of the Asp residue at position 24 may yield a tetrameric SOD3 isoform [55]. This mutant isoform may correspond to the smaller, high molecular weight band at 140 kDa. These findings were further confirmed by measuring SOD3 immunoreactivity in the cerebral cortex. Immunohistochemical analysis revealed higher levels of SOD3 in the ipsilateral tissue of sham rats compared to the ipsilateral cortex of PBS-treated rats. The higher level of staining is most likely due to the higher number of viable neural cells in the cortex after sham operation compared to MCAO. Cells expressing SOD3 after LIF treatment had a morphology resembling pyramidal neurons, and double-labeling tissue with MAP2 and SOD3 antibodies confirmed neuronal localization of SOD3.

Western blotting and histochemical analysis revealed that SOD3 was upregulated in the ipsilateral, but not contralateral hemisphere at 72 h post-MCAO. The absence of LIF-mediated cellular protection under normoxia reflects previous findings with cultured oligodendrocytes [40]. Decreased LDH release from cultured oligodendrocytes following LIF treatment was not observed under normoxia, but LIF significantly reduced LDH release during OGD [40]. Although LIF is transported into the brain via an ATP-dependent carrier [56], Pan et al. demonstrated that LIF transport increases following neuronal injury [57]. Breakdown of the BBB following permanent FCI [58] could allow LIF to reach the ipsilateral hemisphere via paracellular transport. In addition, negative feedback mechanisms that regulate PI3K/Akt signaling under physiological conditions are compromised under FCI [59]. Finally, minimal levels of cellular death under normoxia may account for the lack of significant LIF-mediated neuroprotection under normoxic conditions. Despite the overall increase in SOD activity, neither SOD1 nor SOD2 expression was significantly altered from the ipsilateral tissue of PBS-treated rats. The trend towards decreased SOD2 expression in the ipsilateral hemisphere of LIF-treated rats is most likely due to increased activation of Akt. Previous findings show that Akt may downregulate SOD2 expression via inactivation of the transcription factor FoxO3 [60–62]. Significantly higher levels of SOD1 at 72 h post-MCAO were observed after LIF treatment, but only in contralateral tissue. This finding reflects studies where LIF treatment ameliorates the ALS-like symptoms seen in G93A mice [38, 39]. Although Western blotting did not show increased SOD1 in the ipsilateral tissue of LIF-treated rats, ischemic conditions may cause SOD1 to aggregate. These insoluble aggregates would not be detected in the portion of lysates used for Western blot analysis [63].

The answer to the difference between the efficacies of LIF under normoxia vs ischemia may lie in the regulation of LIFR/gp130. Immunohistochemical detection of LIFR in neurons incubated under normoxia showed that LIF could hypothetically trigger protective signaling in the absence of ischemia. Moreover, oligodendrocytes express LIFR under basal conditions [37]. Future studies may be needed to further explain how LIF signal transduction is regulated in neurons. Several groups reported that the termination of LIF signaling is facilitated via the phosphorylation of both receptor subunits [64, 65]. Our immunohistochemical staining reveals high levels of nuclear LIFR in the absence of LIF signaling. Nuclear LIFR was previously seen under physiological conditions in several cell types, including neurons of the dorsal root ganglion. However, injury to the dorsal root ganglion caused LIFR to translocate from the nucleus to the cytoplasm [66, 67]. Endocytosis of LIFR and gp130 has been previously reported by multiple independent groups [68–70]. While the internalization of LIFR may precede its degradation, it is also possible that the majority of LIFR remains in the nucleus until stimulation with LIF promotes its trafficking to the cell membrane.

The increase in SOD3 mRNA and decrease in LDH release/caspase-3 activation following LIF treatment indicates that upregulation of SOD3 is responsible for LIF’s protective effects against OGD. Furthermore, LIF-treated neurons that were previously transfected with SOD3 siRNA showed no significant difference in LDH release and caspase-3 activation compared to PBS-treated neurons transfected with scrambled siRNA. These results indicate that LIF primarily protects neurons against ischemic oxidative damage via SOD3 upregulation. LIF and siRNA treatment yielded a significant overall change among treatment groups when caspase-3 activation was measured, but not LDH. This discrepancy between these results could be due to several factors. LDH release corresponds to the rate of necrosis [71] while caspase-3 cleavage corresponds to apoptosis. Previous research indicates that neurons undergo both processes during FCI [72, 73]. Caspase-3 activation may be detected prior to LDH release, indicating that staining will identify cells with intact membranes that are beginning to undergo programmed death [74]. Moreover, the larger increase in caspase-3 activation may be due to the inhibitory effect of SOD3 and active Akt on caspase-3-mediated apoptosis [61, 75].

Previous data from this lab indicated that injection of LIF improved several outcomes including infarct size, white matter damage, and functional motor skills at 72 h after permanent FCI. In vitro experiments using cultured oligodendrocytes and Prdx4 antibodies confirmed that the ability of LIF to reduce oxidative damage to white matter during stroke is primarily dependent upon its upregulation of Prdx4 through PI3K/Akt signaling [40]. Prdx4, not unlike SOD3, may be secreted into the extracellular environment to scavenge ROS [76, 77]. By neutralizing extracellular ROS, enzymes such as SOD3 and Prdx4 may protect other cells in the parenchyma by preventing further intracellular damage. LIF-mediated upregulation of SOD3 and Prdx4 in the brain may help regulate redox balance after FCI. Since hydrogen peroxide and water are products of SOD-catalyzed reactions, higher SOD3 expression could potentially lead to excess peroxidation of cellular components. Peroxiredoxin family enzymes catalyze the breakdown of hydrogen peroxide to water and oxygen [78–80]. Therefore, increased Prdx4 expression following LIF treatment would prevent the buildup of hydrogen peroxide generated from excess SOD activity.

Although results from in vitro experiments indicate that the neuroprotective effects of LIF are attenuated following siRNA-mediated knockdown of SOD3, this does not necessarily mean that SOD3 upregulation is the only mechanism of in vivo neuroprotection. Unfortunately, there are no selective SOD3 inhibitors that could be administered following MCAO. Due to the lack of tissue-specific knockout rats available, obtaining a neuron-specific SOD3 knockout rat strain for these experiments would be difficult. To demonstrate that induction of Prdx4 was necessary for in vitro oligoprotection, this laboratory used neutralizing antibodies to block Prdx4 activity prior to OGD [40]. It is likely that the improvement in sensorimotor skills and smaller infarct volume after LIF treatment may be attributed to protective mechanisms other than Prdx4 induction. This lab has shown that LIF confers protection in multiple neural cell types during ischemia. Moreover, systemic LIF administration may result in additional protection that is independent of its effects on cell survival. For instance, LIF plays a crucial role in the development of regulatory T lymphocytes and alternatively activated (M2) macrophages/microglia [81, 82]. As a result, LIF may reduce neurodegeneration triggered by the release of inflammatory splenocytes after stroke. Further investigation will be necessary to determine whether its primary neuroprotective mechanism in vivo is dependent upon SOD3.

This laboratory previously measured infarct volume after LIF treatment using Fluoro-Jade staining due to its ability to specifically label dying neurons [40, 83]. Although a lower number of viable neurons in the infarct and penumbra could contribute to a significant reduction in the total area of Fluoro-Jade-positive tissue, the improvement in motor skills among LIF-treated rats indicated that LIF reduced neuronal degradation in addition to protecting oligodendrocytes in vivo [66]. Previous studies indicate that LIF mRNA levels are approximately 30 times higher in astrocytes 6 h after cortical brain injury compared to pre-injury levels. Microglia were also found to have elevated levels of LIF mRNA at this same time point [41]. This upregulation of LIF transcript following injury to the gray matter indicates that LIF may serve as an endogenous protective factor against neuronal injury. However, the magnitude of damage observed after our model permanent FCI indicates that endogenous LIF release is not sufficient to rescue neurons in the penumbra. These data indicate that damage may be reduced via exogenous supplementation of LIF following injury.

Further studies will be needed to determine the mechanism of LIF-mediated upregulation of SOD3 in neurons. Our group and others have demonstrated that LIF controls the expression of protective genes at the transcriptional level [40, 84]. Therefore, LIF may be activating transcription factors downstream of Akt. The reduced infarct size and improved motor skills observed after LIF treatment in previous studies suggest that it may exert pro-survival effects independent of antioxidant enzymes [40]. Nevertheless, the ability to induce protective antioxidant enzymes makes LIF a promising treatment that will protect neural cells against immediate and delayed damage following ischemic stroke.

## Abbreviations

ALS: Amyotrophic lateral sclerosis
BBB: Blood–brain barrier
DAB: 3,3′-Diaminobenzidine
DAPI: 4′,6-Diamidino-2-phenylindole
FCI: Focal cerebral ischemia
gp130: Glycoprotein 130
HUCB: Human umbilical cord blood
LDH: Lactate dehydrogenase
LIF: Leukemia inhibitory factor
LIFR: LIF receptor
MAP2: Microtubule-associated protein 2
MCAO: Middle cerebral artery occlusion
NO: Nitric oxide
NADPH: Nicotinamide adenine dinucleotide phosphate
OGD: Oxygen glucose deprivation
Prdx4: Peroxiredoxin IV
qRT-PCR: Quantitative reverse-transcriptase PCR
ROS: Reactive oxygen species
SOD: Superoxide dismutase
